# Protective Effects of Monoacylglycerol Lipase Inhibition in Rats with Severe Acute Pancreatitis and Its Possible Mechanism

**DOI:** 10.2174/0118715303335207241225091132

**Published:** 2025-01-17

**Authors:** Tong Su, Hongwei Xu, Ruixia Wang, Tong Xiao, Jing Wang, Shulei Zhao

**Affiliations:** 1 Department of Gastroenterology, Shandong Provincial Hospital Affiliated to Shandong First Medical University, 324 Jingwu Weiqi Rd, Jinan, 250021, China;; 2 Department of Infectious Diseases, Shandong Provincial Hospital Affiliated to Shandong First Medical University, 324 Jingwu Weiqi Rd, Jinan, 250021, China

**Keywords:** Endogenous cannabinoid system, MAGL inhibitor, JZL184, SAP, cGMP, signaling pathway

## Abstract

**Background and Aim:**

In the context of gastrointestinal diseases, the role of monoacylglycerol lipase (MAGL) is significant. Therefore, the objective of this study was to examine the protective effects of MAGL inhibition using JZL184 in rat models of severe acute pancreatitis (SAP) and to explore its mechanism.

**Methods:**

In this study, a rat model of SAP was established, and the rats were divided into three groups for treatment: the Control group (CON), the SAP group (SAP), and the SAP group treated with JZL184 (JZL184). The serum levels of amylase (AMS), alanine aminotransferase (ALT), creatinine (Cr), nitric oxide (NO), cyclic guanosine monophosphate (cGMP), and phosphodiesterase (PDE) were measured using enzyme-linked immunosorbent detection kits. The ascites volume was determined using the cotton ball weighing method. The levels of reactive oxygen species (ROS) were detected using the ROS Kit. Additionally, histological tissue changes were assessed through hematoxylin and eosin staining.

**Results:**

The SAP group showed increased levels of AMS, ALT, Cr, ROS, and ascites volume compared to the CON group. Additionally, the SAP group exhibited congested and edematous lung and pancreatic tissues with inflammation. However, the JZL184 group, when compared to the SAP group, showed decreased levels of AMS, ALT, Cr, and ROS, reduced ascites volume, and significantly reduced lung tissue and pancreatic histopathology scores. In the NO/cGMP/PDE system, compared with the CON group, the levels of NO and PDE in the SAP group were higher and the levels of cGMP were lower. Compared with the SAP group, the JZL184 group decreased NO and PDE levels and increased cGMP levels.

**Conclusions:**

Indeed, the inhibition of MAGL with JZL184 has been found to have a protective effect on rats with SAP. Specifically, it has shown significant improvement in the pathological damage of lung and pancreatic tissues. Furthermore, JZL184 has also exhibited protective effects on the liver and kidney. The mechanism may be related to the effect of JZL184 on the NO/cGMP/PDE signaling pathway.

## 1 INTRODUCTION

Acute pancreatitis (AP) is an unpredictable and potentially fatal disease, with a prognosis that is mainly dependent on the development of organ failure and secondary infection of the pancreas or peripancreatic necrosis [1]. Over the past 10 years, the treatment of acute pancreatitis has evolved towards a multidisciplinary, tailored, and minimally invasive approach, with severe acute pancreatitis (SAP) remaining associated with high mortality despite improvements in treatment and intensive care [2].

SAP can lead to damage in multiple organs, with lungs and intestines being particularly susceptible [3, 4]. MAGL is an enzyme that plays a role in gastrointestinal diseases and breaks down a compound called 2-arachidonoylglycerol (2-AG), which is part of the body's endogenous cannabinoid system [5]. Studies have suggested that 2-AG may be involved in the development of SAP [5-7]. Inhibition of MAGL has been shown to modulate the inflammatory response and protect the intestinal barrier in previous studies [8]. JZL184, a piperidine carbamate that preferentially and irreversibly inhibits MAGL, was the first pharmacological drug to acutely increase 2-AG levels in the brain without changing the levels of anandamide [9]. Thus, JZL184 is the preferred selective MAGL inhibitor and has been observed to have anti-inflammatory, anticancer, antihyperalgesia, and neuroprotective effects [10]. Our own research [11] demonstrated that inhibiting MAGL improved intestinal mucosal barrier injury in rats with SAP. This inhibition also led to significant changes in gene expression and alternative splicing events.

In the NO/cGMP/PDE signaling pathway, the accumulation of cyclic guanosine monophosphate (cGMP) has been reported to have anti-inflammatory and protective effects on the human body [12]. However, the role of the cGMP pathway in SAP has not previously been studied with JZ184 inhibitors. The purpose of this current study is to further investigate the protective effects of MAGL inhibition in rats with SAP and to explore its mechanism. By exploring these effects, we hope to gain a better understanding of the potential benefits of targeting MAGL as a treatment strategy for patients with SAP.

## 2 MATERIALS AND METHODS

### 2.1 Animals

Male Sprague-Dawley (SD) rats weighing 200-230 g were procured from the Experimental Animal Center of Shandong University in Jinan, Shandong, China. The rats were housed in a temperature-controlled animal facility set at 22°C and followed a programmed 12-hour light/dark cycle. All animal experiments were carried out in compliance with the guidelines and regulations established by the Shandong Provincial Hospital Committee on the Use and Care of Animals. The researchers who conducted this study were unaware of the specific information regarding the treatments given to the animals.

### 2.2 SAP Rat Model Establishment

The procedures for creating the SAP rat model and conducting the JZL184 treatment experiments were carried out in accordance with the methodology described in our previous study [11]. SD rats, aged 6-8 weeks, were anesthetized and a median incision was performed to locate the duodenal opening. A No.5 puncture needle was utilized to retrogradely puncture the pancreaticobiliary duct near the duodenal opening. The puncture needle at the duodenal opening and the hilar opening of the common bile duct was secured in place with two non-traumatic artery clamps. A micropump was used to inject 3% sodium taurocholate solution into the pancreatic duct at a uniform rate of 0.1 ml/min (at a dose of 0.1 ml/100 g). The needle was kept in place for 5 minutes after injection. After visually confirming that the color of the pancreas had turned dark red, the puncture needle was removed, the vascular clamp was loosened, and the duodenal wall puncture was sutured to prevent intestinal fluid leakage into the abdominal cavity. After confirming the absence of active bleeding, the abdominal cavity was closed in a layered manner.

### 2.3 Animal Grouping and Sample Collection

The SD rats housed in the facility were equally and randomly divided into three groups (n= 3 each) 24 hours after the surgery: the Control group (CON), the SAP group (SAP), and the SAP treated with JZL184 group (JZL184). The JZL184 group received an intraperitoneal injection of the MAGL inhibitor JZL184 (Cayman Europe, Talin, Estonia). The inhibitor was prepared in a mixture of saline/ethanol/Tween-80 and administered at a dosage of 10 mg/kg. At the same time, in the Control and SAP groups, the SD rats were intraperitoneally injected with an equivalent volume of vehicle. After 24 hours of intervention, the rats were killed for further experiments. We collected blood samples from the arterial blood and centrifuged them at 3,000 g at 4°C for 5 min. The supernatant of blood samples was then stored at −20°C for subsequent serum analysis.

### 2.4 Main Experimental Reagent

The main experimental reagents are given in Table **S1**.

### 2.5 AMS, ALT, and Cr Measurements

Arterial blood samples were obtained 24 hours post-treatment using the manufacturer's instructions as a guide. The optical density (OD) values were measured using a microplate reader at a wavelength of 450 nm, and the concentrations of AMS, ALT, and Cr were calculated and documented accordingly. Each measurement was performed in triplicate to ensure accuracy and consistency.

### 2.6 NO, cGMP and PDE Measurements

Arterial blood samples were obtained 24 hours post-treatment using the manufacturer's instructions as a guide. The optical density (OD) values were measured using a microplate reader at a wavelength of 450 nm, and the concentrations of NO, cGMP, and PDE were calculated and documented accordingly. Each measurement was performed in triplicate to ensure accuracy and consistency.

### 2.7 Determination of ROS Levels

Arterial blood samples were collected 24 hours after treatment. All steps and instructions provided by the manufacturer were followed during the collection and handling of the samples. The fluorescence of the ROS products was measured using various instruments such as a fluorescence spectrophotometer and a fluorescence microplate reader. The measurements were carried out at or near the excitation wavelength of 488 nm and the emission wavelength of 520 nm. To ensure accuracy and consistency, the measurements were performed in triplicate.

### 2.8 Ascites Volume Measurement

After a 24-hour period of creating the model, the abdominal region of the rat was surgically opened. To measure the amount of ascites present in the rat, a dry cotton ball was used to absorb the fluid. The ascites volume was determined by weighing the wet cotton ball and subtracting the weight of the dry cotton ball.

### 2.9 Histopathological Evaluation

The lung tissues and pancreas tissues were preserved in a solution of formalin (4%) to fix them. After fixation, the tissues were embedded in paraffin, allowing for histological analysis. Tissue sections of 3 μm thickness were prepared and stained with hematoxylin and eosin (HE). Using a light microscope, we examined the tissue sections to observe any morphological changes. To assess the extent of injury in the lung and pancreas tissues, we referred to established criteria from previous studies [13, 14]. These criteria include specific parameters or characteristics that allow for a standardized scoring system to determine the degree of injury in the tissues.

### 2.10 Statistical Analysis

The statistical analysis for the data was carried out using R version 4.2.3 software. The experiments were repeated three times to ensure the reliability and consistency of the results. To compare the statistical significance between the two groups, we employed the Student's t-test. The t-test is a widely used statistical test to determine if there is a significant difference between the means of two groups. A *p*-value of less than 0.05 (*p* < 0.05) was considered as indicating a significant variation.

## 3 RESULTS

### 3.1 JZL184 Intervention Decreased AMS, ALT, Cr, and ROS Levels in SAP Rats

Analysis revealed that compared to the CON group, the SAP group and JZL184 group had increased levels of AMS, ALT, Cr, and ROS (*P* < 0.05). Moreover, compared to the SAP group, the JZL184 group showed decreased levels of AMS, ALT, Cr, and ROS with significant differences (*P* < 0.05). These findings are illustrated in Figs. (**1A**-**D**).

### 3.2 JZL184 Intervention Decreased Ascites Volume in SAP Rats

**Fig. (2):**
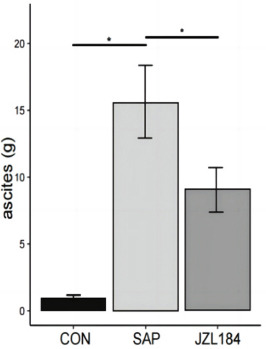
Influence of MAGL inhibitor on ascites volume of rats with SAP. Ascites volume detected in each group. The SAP group had more ascites volume than the CON group (*p* < 0.05). JZL184 reduced ascites volume. n = 3 in each group. *P < 0.05, **p < 0.01, ***p < 0.001.

Analysis showed that compared with the CON group, ascites volume increased in the SAP group and the JZL184 group (*P* <0.05); Compared with the SAP group, ascites volume was reduced in the JZL184 group, with a significant difference (*P* <0.05) (Fig. **2**).

### 3.3 JZL184 Intervention Improved Lung Injury in SAP Rats

**Fig. (3):**
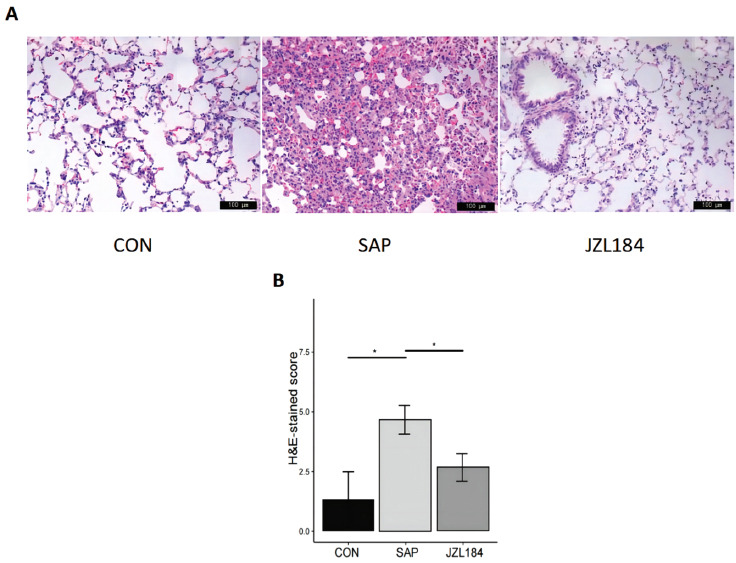
Influence of MAGL inhibitor on lung tissues morphological changes of rats with SAP. (**A**) Representative HE-stained images of lung tissues in each group (original magnification, x200). No obvious lung tissues injury was found in the CON group. However, there were signs of lung tissues injury in the SAP group; (**B**) Pathological scores for the Iung tissues in each group. The lung tissues of SAP rats treated with JZL184 displayed a lesser degree of morphological changes and lower pathological scores than those of the SAP group (*p* < 0.05). n = 3 in each group. *p < 0.05, **p < 0.01, ***p < 0.001.

The results of HE staining revealed that no obvious lung injury was found in the CON group. As shown in Fig. (**3A**), there were signs of lung injury in the SAP group, which included wider alveolar interstitium, edema, hemorrhage, and an increase in inflammatory cells. The lung tissues of SAP rats treated with JZL184 displayed a lesser degree of morphological changes than those of the SAP group (*p* < 0.05) (Fig. **3B**).

### 3.4 JZL184 Intervention Improved Pancreatic Injury in SAP Rats

**Fig. (4):**
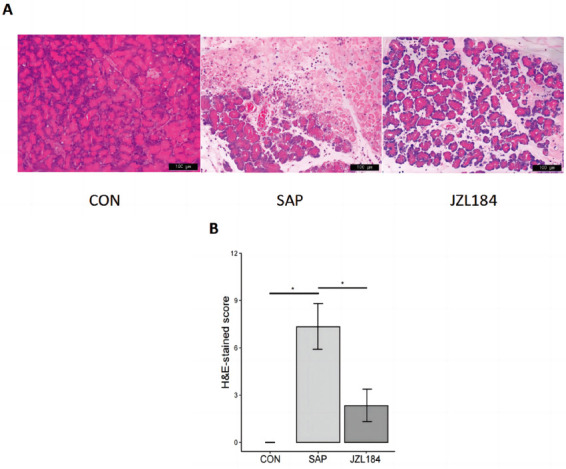
Influence of MAGL inhibitor on pancreatic tissues morphological changes of rats with SAP. (**A**) Representative HE-stained images of pancreatic tissues in each group (original magnification, x200). No obvious pancreatic injury was found in the CON group. However, there were signs of pancreatic injury in the SAP group; (**B**) Pathological scores for the pancreatic tissues in each group. The pancreatic tissues of SAP rats treated with JZL184 displayed a lesser degree of morphological changes and lower pathological scores than those of the SAP group (*p* < 0.05). n=3 in each group. *p < 0.05, **p < 0.01, ** *p* < 0.001.

Upon HE staining, no obvious pancreatic injury was found in the CON group. As shown in Fig. (**4A**), there were signs of pancreatic injury in the SAP group, which included acinar necrosis, hemorrhage, fat necrosis, and an increase in inflammatory cells. The pancreatic tissues of SAP rats treated with JZL184 also displayed a lesser degree of morphological changes than those of the SAP group (*p* < 0.05) (Fig. **4B**).

### 3.5 JZL184 Intervention Affected the NO/cGMP/PDE Signaling Pathway

Analysis revealed that compared to the CON group, the SAP group had increased levels of NO and PDE (*P* < 0.05), and decreased levels of cGMP (*P* < 0.05). Moreover, compared to the SAP group, the JZL184 group showed decreased levels of NO and PDE, and increased levels of cGMP. These findings are illustrated in Figs. (**5A**-**C**).

## 4 DISCUSSION

Identification of MAGL activity was first described in 1966 [15]. This enzyme degrades 2-AG, which is the most abundant endocannabinoid in the body [16]. Notably, MAGL knockout mice have been shown to exhibit strong upregulation of 2-AG [17]. The latest reports suggest the possible involvement of the endogenous cannabinoid system in the mechanism of SAP pathogenesis [18]. MAGL is currently considered a promising therapeutic target for the treatment of numerous diseasesincluding gastrointestinal disorders, cancer, and neurodegenerative and inflammatory diseases [19]. In this study, we established an SAP rat model in order to investigate the protective effect and action mechanism of MAGL inhibition in SAP rats.

SAP development is associated with the release of inflammatory factors like IL-6, TNF-α by pancreatic enzymes, macrophages, and vascular endothelial cells [20]. These factors will not only undermine pancreatic tissues but also give rise to systemic inflammation, resulting in the failure of lung tissues, liver tissues, kidney tissues and other organ tissues [21].

SAP is a condition in which pancreatic tissues experience extensive inflammation and tissue death, leading to rapid disease progression [21]. In this study, it was found that the use of JZL184, a MAGL inhibitor, significantly improved the AMS in rats with SAP compared to the SAP group alone. The pathologic examination further revealed that the JZL184 group exhibited reduced edema, acinar necrosis, hemorrhage, fat necrosis, and leukocyte infiltration area, resulting in significantly lower pathological scores compared to the SAP group. These findings indicate that JZL184, by inhibiting MAGL, offers significant protection to the pancreatic tissues of rats with SAP and effectively mitigates histopathological damage.

Furthermore, aside from affecting pancreatic tissues, lung tissues are highly likely to be involved in the development of severe acute pancreatitis (SAP) [3]. In patients, lung injury is primarily characterized by increased serum amylase and infection index, distorted lung tissue structure, edema, and inflammatory infiltration [22]. Lung injury is closely associated with oxidative stress, which triggers the production of numerous reactive oxygen species [23]. The findings of this research indicate that the inhibition of monoacylglycerol lipase (MAGL) by JZL184 effectively decreases the levels of reactive oxygen species, signifying the suppression of oxidative stress response by JZL184. Moreover, the histopathological assessment scores reveal a significant reduction in lung tissue damage in the JZL184 group, demonstrating the notable protective effect of MAGL inhibition on lung injury in SAP rats.

In addition, the JZL184 compound, which inhibits MAGL, showed protective effects on various internal organs. Significantly improved ALT, Cr, and ascites indexes were observed in the JZL184 group compared to the SAP group, indicating the beneficial effects of MAGL inhibition on organs such as the liver and kidneys in SAP rats. Elevated ALT levels are often an early indicator of liver disease severity, even before noticeable symptoms are present. Kidney injury in SAP usually occurs after the failure of other organs and can result in mortality rates ranging from 25% to 75% [24]. Studies have shown that the mortality rate among SAP patients with liver failure can be as high as 83% and around 5% of SAP patients experience fulminant liver failure [25]. Therefore, investigating the impact of MAGL inhibition on SAP-related damage may offer new insights for the clinical treatment of non-pancreatic complications associated with SAP [26].

The NO/cGMP/PDE signaling pathway is an important system that plays a harmful role in pancreatitis [27]. NO can activate guanidine cyclase that converts GTP to cGMP in this system. On the other hand, NO can also participate in oxidative stress and produce reactive oxygen species, which further leads to oxidative damage [28]. PDE superfamily is cyclic nucleotide that degrades cGMP to 5′-GMP, which is its relative inactive isomer [27]. It has been reported that in pancreatitis, upregulated NO and PDE, on the one hand, can lead to oxidative stress damage, and on the other hand, increased PDE leads to increased cGMP degradation, thereby increasing inflammatory damage [27]. In this study, MAGL inhibitor JZL184 can increase the content of cGMP and improve the above reaction caused by SAP, which not only improves the damage of pancreas and lung tissue but also has a significant protective effect on all organs of rats.

The research team has conducted extensive preliminary experiments on the role of MAGL inhibition. We discovered that in rats with SAP, the MAGL inhibitor JZL184 provided protection against intestinal permeability injury [11]. Through transcriptome sequencing, it was observed that JZL184 treatment possibly reduces the expression of cell focal adhesions and the PI3K-Akt signaling pathway, while also influencing the alternative splicing of genes via modulation of RNA binding protein expression.

There are some limitations of our approach in this study. Firstly, unlike previously published studies on the endocannabinoid system, the causal relationship between the pathway we investigated and the pathogenesis of SAP, as well as the effects of JZL184, remains unclear. Secondly, we did not conduct any further genetic research. Therefore, we will conduct further research on relevant aspects in the future to supplement and explain the current conclusions. Due to the constraints of experimental conditions, the number of rats included in this study was limited. Consequently, this limitation partially diminished the persuasive power of the obtained data.

## CONCLUSION

The findings imply that inhibiting MAGL with JZL184 offers protective effects on various organs in SAP rats, particularly on lung and pancreatic tissues, thereby ameliorating tissue damage. Furthermore, MAGL inhibition noticeably improved their pathological condition. The mechanism may be related to the effect of JZL184 on the NO/cGMP/PDE signaling pathway. Nonetheless, to fully comprehend the mechanism, additional *in vivo* and *in vitro* experiments are necessary to investigate the protective impact of MAGL inhibition on pancreatic tissue and the entire body.

## Figures and Tables

**Fig. (1):**
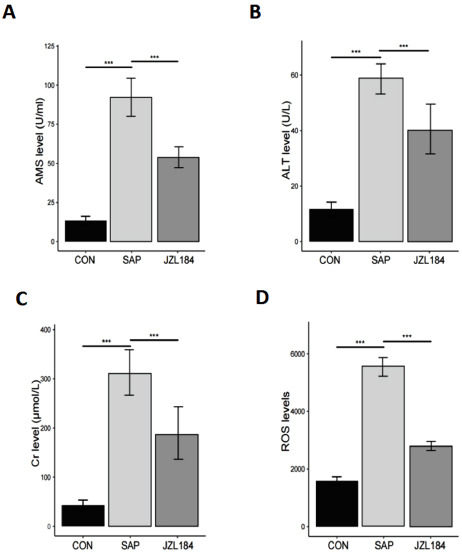
nfluence of MAGL inhibitor on various organs of rats with SAP. (**A**) AMS levels detected in each group. The levels of AMS were higher in the SAP group than in the CON group (*p* < 0.001). JZL184 decreased AMS levels; (**B**) ALT levels detected in each group. The levels of ALT were higher in the SAP group than in the CON group (*p* < 0.001). JZL184 decreased ALT levels; (**C**) Cr levels detected in each group. The levels of Cr were higher in the SAP group than in the CON group (*p* < 0.001). JZL184 decreased Cr levels; (**D**) ROS levels detected in each group. The levels of ROS were higher in the SAP group than in the CON group (*p* < 0.001). JZL184 decreased ROS levels. n = 3 in each group. *p < 0.05, **p < 0.01, ***P < 0.001.

**Fig. (5):**
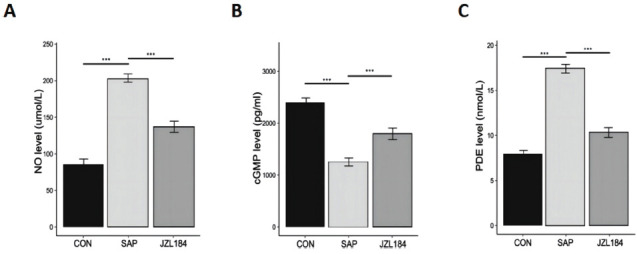
Influence of MAGL inhibitor on the NO/cGMP/PDE signaling pathway of rats with SAP. (**A**) NO levels detected in each group. The levels of NO were higher in the SAP group than in the CON group (*p* < 0.001). JZL184 decreased NO levels; (**B**) cGMP levels detected in each group. The levels of cGMP were lower in the SAP group than in the CON group (*p* < 0.001). JZL184 Increased the cGMP levels; (**C**) PDE levels detected in each group. The levels of PDE were higherin the SAP group than in the CON group (*p* < 0.001). JZL184 decreased PDE levels. n=3 in each group. *p < 0.05, **p < 0.01, ***p < 0.001.

## Data Availability

The data and supportive information are available within the article.

## LIST OF ABBREVIATIONS

AP: Acute Pancreatitis
cGMP: Cyclic Guanosine Monophosphate
MAGL: Monoacylglycerol Lipase
NO: Nitric Oxide
OD: Optical Density
PDE: Phosphodiesterase
SAP: Severe Acute Pancreatitis
SD: Sprague-Dawley
