# Supplementary material for: Protective Effects of Monoacylglycerol Lipase Inhibition in Rats with Severe Acute Pancreatitis and Its Possible Mechanism
Source: Endocr Metab Immune Disord Drug Targets. 2025 Jan 17;26:E18715303335207. doi: 10.2174/0118715303335207241225091132 (PMC13284646; doi:10.2174/0118715303335207241225091132)
Supplement: Supplementary file 1 [file EMIDDT-26-E18715303335207_SD1.pdf]

Supplementary Material

Protective Effects of Monoacylglycerol Lipase Inhibition in Rats with Severe Acute Pancreatitis and Its Possible Mechanism

Tong Su[1, #], Hongwei Xu[1], Ruixia Wang[1], Tong Xiao[1], Jing Wang[2,*] and Shulei Zhao[1,*]

[1]*Department of Gastroenterology, Shandong Provincial Hospital Affiliated to Shandong First Medical University, 324 Jingwu Weiqi Rd, Jinan, 250021, China;* [2]*Department of Infectious Diseases, Shandong Provincial Hospital Affiliated to Shandong First Medical University, 324 Jingwu Weiqi Rd, Jinan, 250021, China*

Supplementary Table 1. Main experimental reagent

Experimenta Reagent	Company
Rat Amylase (AMS) ELISA Kit	Bioswamp (Wuhan, China)
Rat Alanine Aminotransferase (ALT) ELISA Kit	Bioswamp (Wuhan, China)
Rat Creatinine (Cr) ELISA Kit	Bioswamp (Wuhan, China)
The α-Amylase (AMS) Kit	Jiancheng (Nanjing, China)
Reactive Oxygen Species (ROS) Kit	BestBio (Shanghai, China)
H-E staining kit	Bioswamp (Wuhan, China)
nitric oxide (NO) ELISA Kit	LaiEr Bio-Tech (Hefei,China)
cyclic guanosine monophosphate (cGMP) ELISA Kit	LaiEr Bio-Tech (Hefei,China)
phosphodiesterase (PDE) ELISA Kit	LaiEr Bio-Tech (Hefei,China)
